# Selective Carbon Dioxide Hydrogenation to Olefin-Rich Hydrocarbons by Cu/FeOx Nanoarchitectures Under Atmospheric Pressure

**DOI:** 10.3390/nano15050353

**Published:** 2025-02-24

**Authors:** Muhammad I. Qadir, Naděžda Žilková, Libor Kvítek, Stefan Vajda

**Affiliations:** 1 Department of Nanocatalysis, J. Heyrovský Institute of Physical Chemistry, Dolejškova 2155/3, 18223 Prague 8, Czech Republic; 2 Department of Physical Chemistry, Faculty of Science, Palacký University Olomouc, 17. Listopadu 12, 77146 Olomouc, Czech Republic

**Keywords:** carbon dioxide hydrogenation, Cu/FeOx, olefins, nanocatalyst

## Abstract

The conversion of carbon dioxide into fuels and fine chemicals is a highly desirable route for mitigating flue gas emissions. However, achieving selectivity toward olefins remains challenging and typically requires high temperatures and pressures. Herein, we address this challenge using 12 nm copper nanoparticles supported on FeOx micro-rods, which promote the selective hydrogenation of CO_2_ to light olefins (C_2_–C_4_) under atmospheric pressure. This catalyst achieves up to 27% conversion and 52% selectivity toward C_2_–C_4_ olefins, along with the production of C_2_–C_4_ paraffins, C_5_+ hydrocarbons (with all C_1+_ products totalling to up to about 75%), and methane, while suppressing CO formation to just 1% at 340 °C. The enhanced performance of the Cu/FeOx pre-catalyst is attributed to the efficient in situ generation of iron carbides (Fe_5_C_2_) in the presence of copper nanoparticles, as confirmed by ex situ XRD analysis. Copper facilitates the reduction of FeOx to form Fe_5_C_2_, a crucial intermediate for shifting the reaction equilibrium toward higher hydrocarbons. The hydrogenation of CO_2_ to higher hydrocarbons proceeds through the reverse water–gas shift reaction coupled with Fischer–Tropsch synthesis.

## 1. Introduction

The conversion of carbon dioxide is one of the major challenges of modern times due to the increased concentration of this gas in the atmosphere, which is driven by contemporary industrial activities and is a significant contribution to global warming [1]. A highly desirable approach to address this challenge is the conversion of CO_2_ into C_1_ feedstocks such as carbon monoxide, methanol, and methane, or into longer-chain hydrocarbons and fuels. Recently, several breakthroughs in CO_2_ hydrogenation have been reported, enabling the production of valuable chemicals such as carbonates, aldehydes, amides, and esters [2,3,4,5,6], including new emerging catalytic systems [7]. While the electrochemical reduction of CO_2_ also yields C_1_ chemicals like carbon monoxide, methane, methanol, and formic acid, this process is still far from widespread implementation [8,9]. When it comes to the production of higher hydrocarbons, the thermal activation of CO_2_ and H_2_ on a catalyst surface is considered one of the most promising routes [10]. This process involves the initial conversion of CO_2_ to CO via the reverse water–gas shift (RWGS) reaction, followed by the conversion of the resulting CO into higher hydrocarbons through Fischer–Tropsch (FT) synthesis, ultimately yielding clean fuels and lubricants [11].

Designing efficient catalysts for the selective conversion of CO_2_ is challenging, as they must be active for both the RWGS and FT processes. The RWGS reaction is slightly endothermic, while the FT process is exothermic [12]. Therefore, achieving higher CO_2_ conversion requires the FT pathway to be efficient enough to overcome the thermodynamic limitations of the RWGS reaction [13]. Several catalysts based on Ru, Ir, Fe, and Co have been reported for CO_2_ conversion to higher hydrocarbons, but the search for highly selective catalysts that produce the desired products remains ongoing. Cobalt-based catalysts are highly active FT catalysts for producing long-chain hydrocarbons from syngas (CO + H_2_). However, when CO_2_ is used as the feedstock, these catalysts predominantly produce methane [14,15], while methane production prevails also for bimetallic catalysts like Co-Fe [16], Ru-Co_3_O_4_, [17] and Co-Pt/TiO_2_ [14], or for precious metal based catalysts made of Ru and Ir, the latter being also prohibitively expensive [18,19].

Iron-based catalysts have gained significant attention for their ability to directly convert CO_2_ into short-chain olefins due to their intrinsic RWGS and FT activities. Several reports have explored alkali-doped (K, Na), Fe-based catalysts for generating C_2_–C_4_ olefins, but these require harsh conditions for pre-treatment under hydrogen or CO atmospheres for extended periods (>300 °C, >12 h) [20,21,22,23,24,25,26,27,28,29,30]. Cu-promoted Fe-based catalysts have also been studied, but they exhibit low selectivity toward C_2_–C_4_ olefins (<25%), with CH_4_ as the main product [31,32,33]. Therefore, the production of higher hydrocarbons from CO_2_ hydrogenation highlights the need for a delicate balance between the RWGS and FT steps. Such a balance can potentially be achieved by using multifunctional catalysts that efficiently drive both the RWGS and FT processes. In this work, we address this challenge by designing a copper–iron oxide (Cu/FeOx) catalyst, which leverages the inherent properties of both Cu and FeOx—Cu activates hydrogen and acts as the RWGS catalyst, while FeOx enhances the production of C_2_–C_4_ olefins via FTS [33,34].

## 2. Experimental Section

All chemicals, including N,N-dimethylacetamide, oxalic acid (hydrated), iron chloride, copper sulfate pentahydrate (CuSO_4_·5H_2_O), and hydrazine hydrate (50–60%), were purchased from Sigma-Aldrich (Prague, Czech Republic). CO_2_ (99.99%) and H_2_ (99.99%) were supplied by Airgas (Plzeň, Czech Republic). XRD experiments were performed using a D/max-3B diffractometer with Co Kα radiation. Scans were conducted in the 2θ range of 5–105° at a scan rate of 10°/min. The individual Fe components were quantified using Rietveld analysis of the XRD data, employing High Score Plus (Malvern Panalytical, Malvern, UK, Version 2021) software and the PDF-4+ and ICSD databases.

### 2.1. Preparation of Cu/FeOx Catalyst

Cu/FeOx nanomaterial was prepared using a modified wet impregnation method, as reported earlier [35]. First, a FeOx micro-rod was prepared by calcinating the iron oxalate at 175 °C for 12 h in air with a 5 °C/min ramp according to the reported method [36]. The obtained FeOx (900 mg) was dispersed in distilled H_2_O (188 mL). Then, 10 mL of an aqueous solution of CuSO_4_·5H_2_O (1.57 mmol) was added slowly. The reaction mixture was sonicated for 2–3 min. The pH of the solution was adjusted to 10 using 1 M NaOH. Next, 3 mL of hydrazine hydrate (50–60%) was added, and the mixture was sonicated for 10 min. The resulting Cu/FeOx was isolated by centrifugation, washed thoroughly with distilled H_2_O, followed by acetone (20 mL) and CH_2_Cl_2_ (20 mL), respectively. The obtained Cu/FeOx was dried at 110 °C for 2 h with a 5 °C/min ramp. The copper content in the Cu/FeOx catalyst was 12.25 ± 1.1 wt %, determined by ICP-MS using an AAS instrument.

### 2.2. Catalytic Tests

All catalytic tests were performed using a Microactivity Reactor System (PID Eng&Tech/Micromeritics, Barcelona, Spain) with a quartz reactor (32 cm in length, 12.7 mm in diameter) in continuous flow mode. Typically, 200 mg of catalyst was placed in the center of the reactor, supported on 20 mg of quartz wool. A K-type thermocouple was inserted into the catalyst bed to monitor the reaction temperature. Initially, the catalyst was conditioned at 250 °C in He (30 mL/min) for 40 min. After conditioning, a mixture of CO_2_/H_2_/He (1/4/3.3, total flow 25 mL/min) was introduced, and an aliquot was taken for GC analysis after 20 min. The temperature was then increased to 280 °C at a rate of 5 °C/min under the gas mixture, and the products were analyzed by GC. The reaction was monitored from 250 to 410 °C in 30 °C intervals, and at each temperature, the outlet gas was analyzed using a GC (Agilent 6890, Santa Clara, CA, USA) equipped with a TCD (HP-PLOT/Q) and FID (Al_2_O_3_/KCl). After collecting data at 410 °C, the temperature was decreased to 250 °C under He (30 mL/min). Then, the same CO_2_/H_2_/He mixture (1:4:3.3, total flow 25 mL/min) was introduced to run a second heating ramp from 250 to 410 °C, and the products were analyzed in 30 °C intervals. To prevent the possible condensation of reaction products, the temperature of the entire pipeline from the reactor to the GC was maintained at 110 °C during the catalytic tests. The reactor pressure was approximately 1 atm. The CO_2_ conversion and products selectivity was determined by the reported methods [37].

## 3. Results and Discussion

### 3.1. Catalysts Synthesis and Characterization

FeOx and Cu/FeOx catalysts were prepared by the wet impregnation method. FeOx micro-rods were prepared by the thermal decomposition of iron oxalate [38] at 175 °C [36]. The Cu/FeOx pre-catalyst was then prepared by decorating the FeOx rods with Cu nanoparticles through the reduction of an aqueous solution of CuCl_2_·2H_2_O with hydrazine hydrate at room temperature in the presence of FeOx rods [35]. The obtained FeOx and Cu/FeOx catalysts were isolated by centrifugation, washed with water and acetone, and dried at 110 °C. The resulting reddish powders were analyzed by transmission electron microscopy (TEM), scanning electron microscopy (SEM), scanning transmission electron microscopy (STEM), and powder X-ray diffraction (XRD).

**Figure 1:**
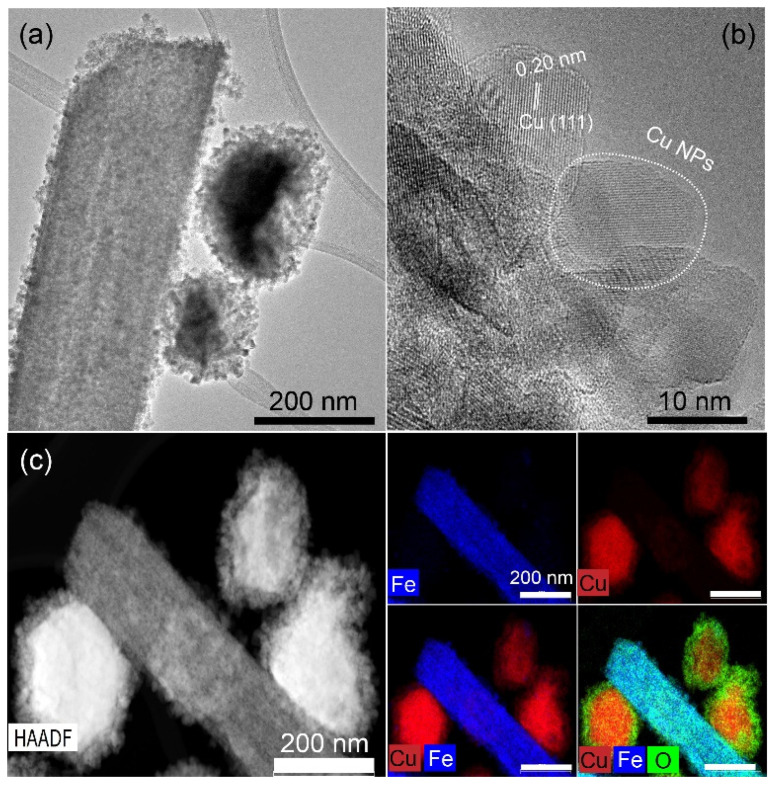
(**a**,**b**) HR-TEM images of as-prepared Cu/FeOx; (**c**) STEM-HAADF of Cu/FeOx and EDS mapping of Fe, Cu and their overlap elements. The bars for EDS mapping images are 200 nm.

The HR-TEM images (Figure 1a,b) of the as-prepared Cu/FeOx show irregularly shaped Cu nanoparticles with a mean diameter of about 12 nm and lattice fringe spacing of 0.20 nm, corresponding to the (111) plane of copper, on the surface of the FeOx rods, which have a width of about 200 nm. The STEM-HAADF images with EDS elemental mapping of Cu and Fe (Figure 1c) show the atomic distribution of Cu, Fe, and O in the sample, revealing a thin oxide layer on the surface of the copper nanoparticles. For the SEM, EDS images, and XRD data of the as-prepared FeOx catalyst, see Figures S1a,b,e,f and S2a, respectively.

**Figure 2:**
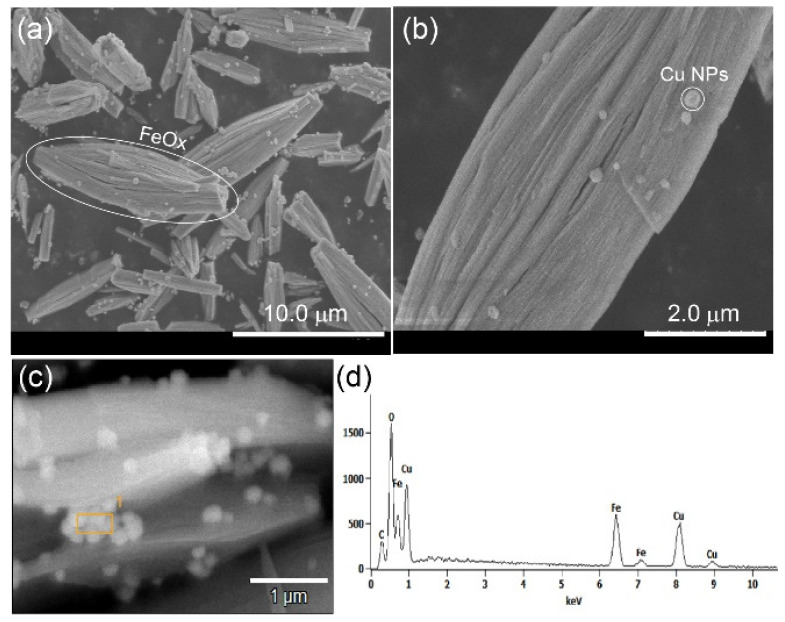
SEM images of as-prepared Cu/FeOx catalyst (**a**–**c**), showing submicrometric structure of the FeOx rods and small nanoparticles of Cu (the orange rectangle with superscript 1 on (**c**)) on their surface. (**d**) EDX of the selected particles. The SEM images of the bare FeOx catalyst shown in Figure S1 reveal similar rods.

The SEM micrographs of Cu/FeOx in Figure 2 show FeOx rods up to about 12 μm in length. Cu nanoparticles were dispersed onto the FeOx rods, as confirmed by EDX (Figure 2d).

**Figure 3:**
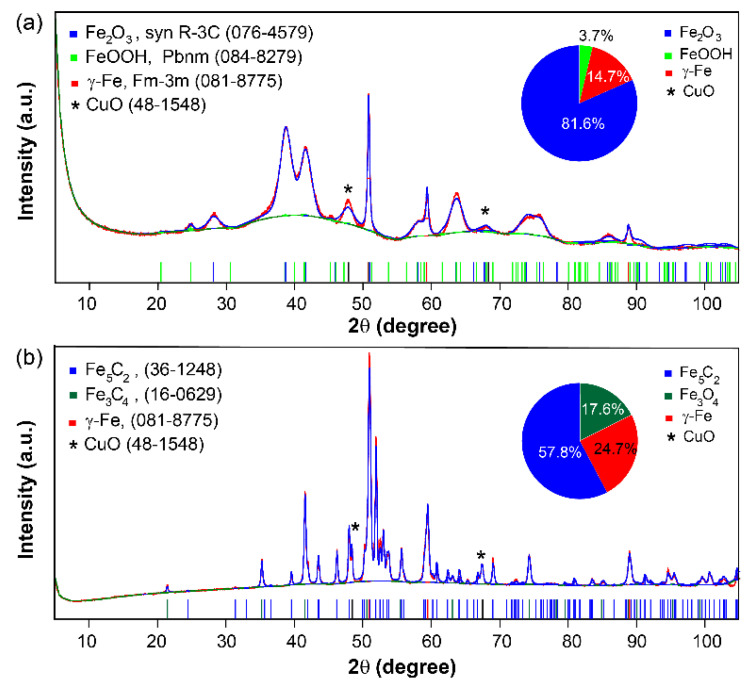
XRD Rietveld refinement analysis of (**a**) as-prepared Cu/FeOx and (**b**) spent Cu/FeOx catalyst.

Rietveld refinement analysis of the XRD pattern of the as-prepared Cu/FeOx catalyst (Figure 3a) revealed that the catalyst contained α-Fe_2_O_3_, β-FeOOH, and γ-Fe at 81.6%, 3.7%, and 14.7%, respectively. The additional diffraction peaks at 48° (202) and 68.4° (220) confirmed the presence of CuO [39].

### 3.2. Catalytic CO_2_ Hydrogenation

**Figure 4:**
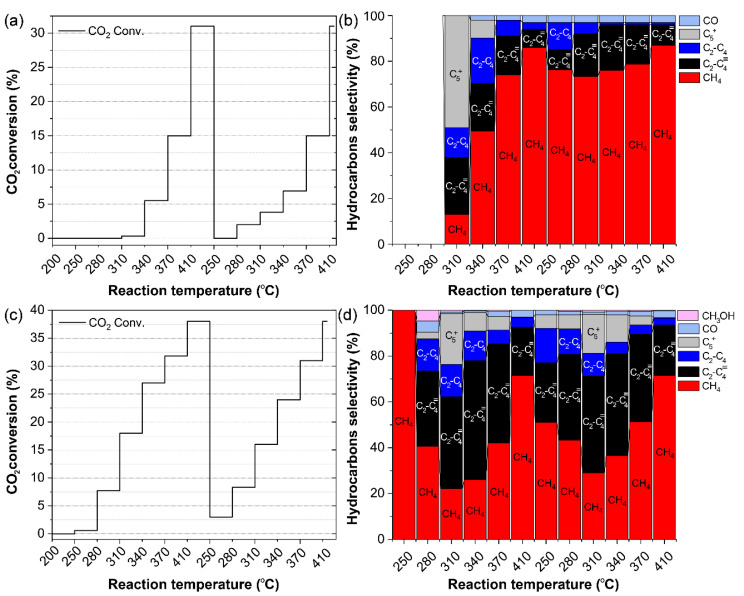
Catalytic hydrogenation of CO_2_ (**a**,**b**) by FeOx and (**c**,**d**) Cu/FeOx nanomaterials. Cat. 200 mg, CO_2_/H_2_/He (1/4/3.3, total flow 25 mL/min, reaction pressure 1 atm). After the first ramp, the catalyst was cooled down under He before starting the second ramp.

The catalytic tests were performed in a fixed-bed flow reactor using 200 mg of catalyst, under a gas mixture consisting of CO_2_, H_2_, and He in a 1:4:3.3 ratio, with a total flow rate of 25 mL/min, across a temperature range of 250–410 °C, applying two consecutive temperature ramps. Note that the pressure in the reactor was approximately 1 atm. The catalysts were used as-prepared, without pre-reduction or any other pre-treatment. Figure 4a,b show the results obtained for the bare FeOx catalyst. During the first temperature ramp, no hydrocarbons were detected at 250 °C or at 280 °C, in contrast to the catalyst being active at 280 °C during the second temperature ramp. At 310 °C, 0.3% CO_2_ conversion was observed, with hydrocarbon selectivities of 13% CH_4_, 13% C_2_–C_4_ paraffins, 25% C_2_–C_4_ olefins, and 49% C_5_+ hydrocarbons, with no CO detected. CO_2_ conversion reached 5.5%, and the selectivity toward C_5_+ hydrocarbons dropped to 8%, accompanied by an increase in CH_4_ selectivity to 50% at 340 °C (Figure 4a). As CO_2_ conversion reached 31% at 410 °C, the selectivity toward higher hydrocarbons significantly decreased, with CH_4_ selectivity rising to 87% (Figure 4b). A similar phenomenon was reported for bulk Fe, which acts as a CO methanation catalyst at higher temperatures (>320 °C) [11,40].

Notably, during the second temperature ramp, significant CO_2_ conversion of 2% and 3.8% was observed at 280 °C and 310 °C, respectively, (Figure 4a). This increase in conversion can be correlated with the formation of partially reduced Fe_3_O_4_ during the first ramp, which has been reported as the active phase for CO_2_ hydrogenation due to its higher CO_2_ chemisorption ability compared to Fe_2_O_3_ [41,42]. XRD analysis (Figure S2) of the spent catalyst confirmed that the morphology of the FeOx catalyst changed from Fe_2_O_3_ to pure Fe_3_O_4_. This change in morphology was previously observed during CO_2_ hydrogenation using Fe_2_O_3_ catalysts [38]. Under identical reaction conditions, Cu NPs alone showed only about 1% CO_2_ conversion with 100% methane selectivity. The comparison of the conversion and selectivity of the FeOx catalyst indicated that the catalyst evolved during the first temperature ramp, reaching its final state at 370 °C during the first ramp. The catalyst produced primarily methane, with up to about 20% higher hydrocarbons and with the olefin fraction increasing with temperature.

The introduction of Cu to FeOx proved to be crucial in improving both CO_2_ conversion and selectivity toward olefins. As reported for iron-based catalysts, these maintain a low CO coverage during CO_2_ hydrogenation, which leads to low CO and high H_2_ adsorption on the catalyst surface, resulting in a large amount of methane production [16]. Copper as an effective RWGS catalyst [43], in combination with iron, can lead to higher CO surface coverage and subsequently increased C_2_+ selectivity through the synergistic function of the two components of the Cu/FeOx catalyst. This synergy can also promote olefin production, which is understood to result from the higher hydrogenation barrier of CH_2_ compared to those for C−C bond formation and CH−CH conversion to olefins [44]. Similar outcomes were observed during CO_2_ hydrogenation by a bimetallic Cu-Fe alloy catalyst [33]. The observed performance of the Cu/FeOx catalyst toward olefin production compares very favorably with that reported for other Fe-based catalysts (see Table S1 for comparison from the literature), except for Na-FeOx [45], which exhibits 64% C_2_–C_4_ selectivity toward olefins. However, this comes at the expense of an order of magnitude higher CO production, and a reaction pressure of 3.0 MPa, which are much harsher conditions than those used for the Cu/FeOx catalyst reported here. The high selectivity toward olefins by the Cu/FeOx catalyst can be correlated to the generation of iron carbide (Fe_5_C_2_), the known active catalytic species for CO_2_ hydrogenation [46], as confirmed by the XRD of the spent Cu/FeOx catalyst (Figure 3b), which shows the presence of 57.8% Fe_5_C_2_, in addition to 17.6% Fe_3_O_4_ and 24.7% γ-Fe. These Fe components were previously detected when the catalyst with the similar composition was used for CO_2_ hydrogenation to CO [35].

**Figure 5:**
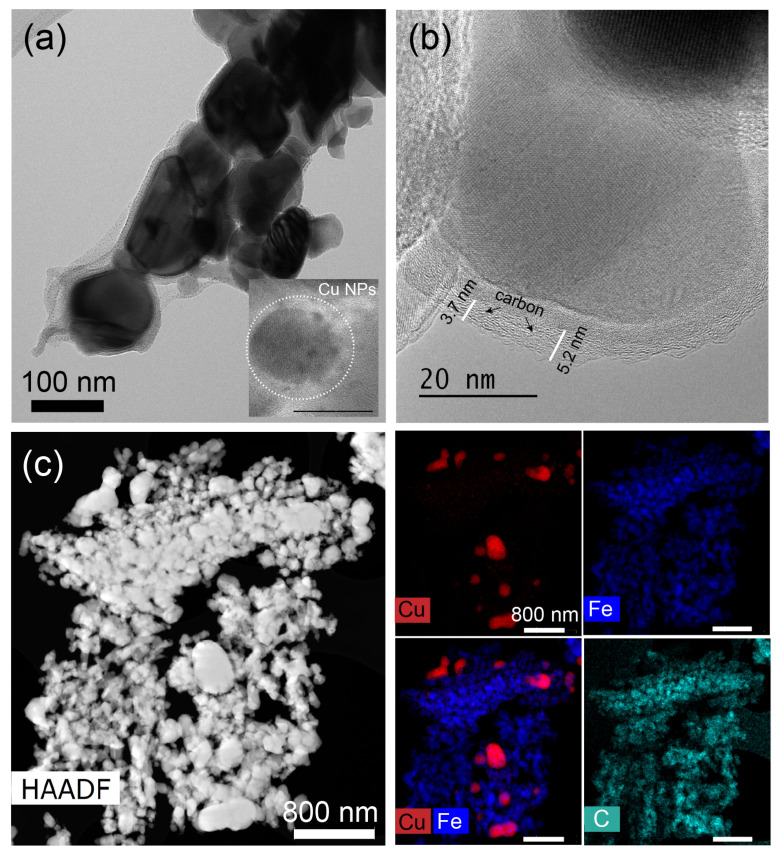
(**a**,**b**) HR-TEM images of spent Cu/FeOx, and (**c**) STEM-HAADF and EDS mapping of Fe, Cu and C of the spent Cu/FeOx catalyst after 10 h. The bar in inset of Figure (**a**) is 20 nm, whereas the EDS mapping images have an 800 nm bar.

After completion of the second temperature ramp, the Cu/FeOx catalyst was cooled down to 340 °C in He, and a 10 h test was performed at this temperature (Figure S3). During this time on stream, CO_2_ conversion marginally decreased by about 4%; however, the selectivity between C_2_–C_4_ olefins and CH_4_ flipped. The CH_4_ selectivity increased from 26% to 55.2%, while the selectivity for C_2_–C_4_ olefins dropped from 52% to 32%. We hypothesize that this change in selectivity could be caused by the accumulation of carbon on the catalyst surface. HR-TEM revealed that the spent catalyst was encapsulated in a carbon shell with a thickness of 3–5 nm, as confirmed by EDS elemental mapping (Figure 5), a deposition of carbon that has been reported for iron-based catalysts in various reactions [47,48,49,50]. The morphology of the spent Cu/FeOx catalyst remained rod-like, as shown in Figure S4.

Recently, it was reported that differently shaped Cu/FeOx catalysts for CO_2_ hydrogenation from 250 to 410 °C exhibited higher selectivity toward CH_4_, ranging from 70% to 100%, where controlling the morphology of the iron oxides was challenging [51]. The morphology of the iron oxide changes from a rod-like to a wool-like structure with an increase in copper precursor. To examine the effect of controlled iron oxide shapes with and without Cu, we were able to preserve the rod-like shape of the iron oxide catalyst in the presence of the copper precursor by using NaOH, which stabilizes the surface morphology and size of the nanomaterials [52,53]. When comparing the morphology of our 12% Cu/FeOx catalyst with the reported 1%, 3%, and 5% Cu/FeOx catalysts (Figures S6 and S7), our iron oxide successfully preserved its rod-like shape. Moreover, the reported catalysts contained 51–55% α-FeOOH species (Table S2), which require relatively high reduction temperatures compared to Fe_2_O_3_. In contrast, our Cu/FeOx catalyst contains about 82% Fe_2_O_3_, while pristine FeOx is 100% Fe_2_O_3_. The higher CO_2_ conversion and selectivity toward higher hydrocarbons observed with our catalysts are related to the presence of a higher amounts of Fe_2_O_3_ (81.6%) and copper (12%). The higher amount of Cu can more effectively reduce Fe_2_O_3_ to catalytically active Fe(0) and Fe_5_C_2_ intermediates [54]. The higher the amount of generated Fe_5_C_2_, the greater the polymerization of carbon intermediates into long-chain hydrocarbons. Post-XRD analysis of Cu/FeOx showed the presence of 54.7% Fe_5_C_2_ (Figure 3b).

In summary, iron carbides are considered active catalytic species that induce chain propagation via Fischer–Tropsch synthesis (FTS) to yield higher hydrocarbons. The chemical and morphological changes in the Cu/FeOx pre-catalyst have significant effects on catalytic activity and selectivity toward higher hydrocarbons. The improved performance of the Cu/FeOx catalyst is attributed to the efficient in situ generation of iron carbide (Fe_5_C_2_) in the presence of Cu nanoparticles, which promote the reduction of iron oxide through H-spillover hydrogen [54]. This may represent the first step in converting iron into iron carbide by carbonation of the reduced metallic iron. In contrast, when the bare FeOx catalyst was used, no Fe_5_C_2_ formation was observed (Figure S2, confirmed by XRD), and CH_4_ was the main product (Figure 4b). This highlights the crucial role of Cu. The copper component not only activates hydrogen and promotes the reduction of FeOx, but it also facilitates the reverse water–gas shift (RWGS) reaction to generate CO, which then enters the FT pathway driven by Fe [55,56]. It is presumed that CO is activated on the surface of iron carbides, followed by sequential hydrogenation to CH, CH_2_, CH_3_, and eventually CH_4_, or it competes to form C_2_+ hydrocarbons [44,57].

## 4. Conclusions

A catalyst consisting of Cu nanoparticles supported on FeOx was prepared using the wet impregnation method. The catalyst demonstrated a 27% CO_2_ conversion with 52% selectivity toward C_2_–C_4_ olefins at 340 °C under atmospheric pressure, with suppressed CO formation. The catalytic performance of the Cu/FeOx pre-catalyst is relayed on the in situ generated iron carbides, formed through the reduction of FeOx to iron carbides and driven by Cu, crucial intermediates for shifting the reaction equilibrium toward higher hydrocarbons. However, after 10 h on stream at 340 °C, the catalyst’s activity decreased by 4%, and the selectivity toward C_2_–C_4_ olefins declined from 52% to 32%, highlighting the need for further improvement in the catalytic materials. Despite this, the findings from this study suggest a promising pathway for efficiently converting carbon dioxide into olefins under atmospheric pressure using noble-metal-free catalysts.

## Data Availability

Data is contained within the article or Supplementary Materials.

## Supplementary Materials

The following supporting information can be downloaded at https://www.mdpi.com/article/10.3390/nano15050353/s1: Figure S1. SEM images of as prepared FeOx. Figure S2. PXRD pattern of the as prepared FeOx catalyst. Figure S3. Stability test of the Cu/FeOx catalyst. Figure S4. SEM images of the spent Cu/FeOx catalys. Figure S5. TEM images of the fresh FeOx spent catalyst. Figure S6. SEM images of the studied FeOx and 12%-Cu/FeOx catalysts compared with previously reported catalyst with different Cu loading. Figure S7. TEM images of the studied 12%-Cu/FeOx catalyst and a previously reported 5%-Cu/FeOx catalyst. Table S1. Literature review of Fe-based catalysts for CO_2_ hydrogenation. Table S2. XRD analyses comparison of the studied catalysts with previously reported ones.
