# Supplementary material for: Selective Carbon Dioxide Hydrogenation to Olefin-Rich Hydrocarbons by Cu/FeOx Nanoarchitectures Under Atmospheric Pressure
Source: Nanomaterials (Basel). 2025 Feb 24;15(5):353. doi: 10.3390/nano15050353 (PMC11901991; doi:10.3390/nano15050353)
Supplement: Supplementary file 1 [file nanomaterials-15-00353-s001.zip › nanomaterials-3423544-supplementary.pdf]

Supporting Material

Atmospheric Pressure Selective Carbon Dioxide Hydrogenation to Olefin-rich Hydrocarbons by Cu/FeOx Nanoarchitectures

Muhammad I. Qadir,*[1] Naděžda Žilková,[1] Libor Kvítek[2] and Stefan Vajda*[1]

[1] Department of Nanocatalysis, J. Heyrovský Institute of Physical Chemistry v.v.i., Czech Academy of Sciences, Dolejškova 2155/3, 18223 Sciences, Dolejškova 2155/3, 18223 Prague 8, Czech Republic

[2] Department of Physical Chemistry, Faculty of Science, Palacký University Olomouc, 17. listopadu 12, 77146 Olomouc, Czech Republic

Table of contents

Content	Page
1. SEM images of the FeOx catalyst before and after CO_2 hydrogenation	S2
2. PXRD pattern of the FeOx catalyst before and after CO_2 hydrogenation	S3
3. Stability test of the Cu/FeOx catalyst for CO_2 hydrogenation	S3
4. SEM images of the Cu/FeOx catalyst after CO_2 hydrogenation	S4
5. TEM images of the FeOx catalyst before and after CO_2 hydrogenation	S4
6. Literature comparison of CO_2 hydrogenation to hydrocarbons by different Fe-based catalysts	S5
7. Morphology and XRD comparison of the composition of the Cu/FeOx catalyst with previously reported catalysts	S6
8. References	S7

1. SEM images of the FeOx catalyst before and after CO_2 hydrogenation

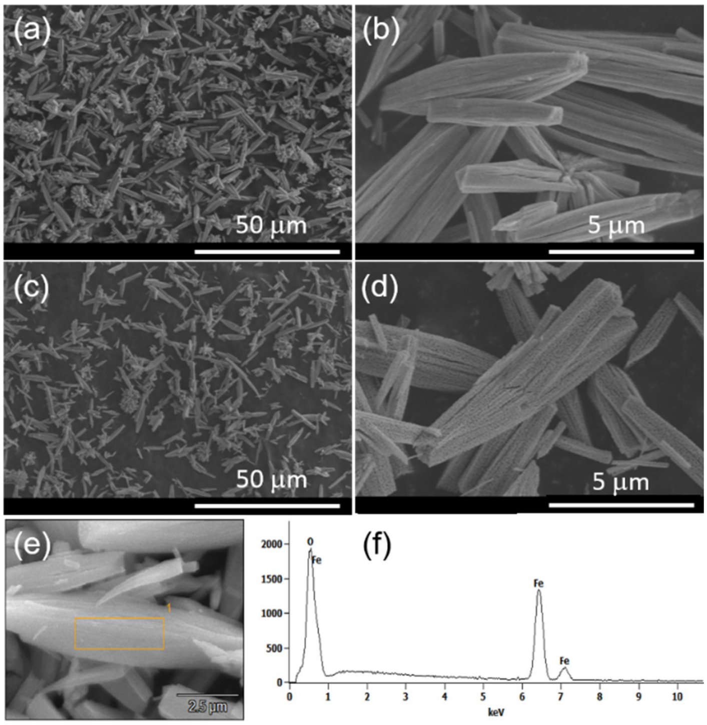

Figure S1. SEM images (a,b) and EDX (e,f) of as prepared FeOx. SEM images (c,d) of spent FeOx catalyst revealed that the catalyst maintained its rod like morphology.

2. PXRD pattern of the FeOx catalyst before and after CO_2 hydrogenation

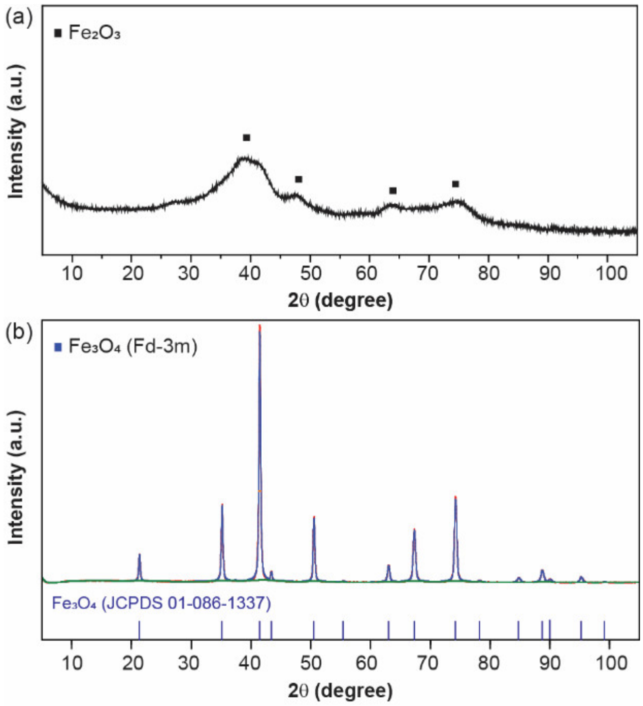

Figure S2. XRD analysis of FeOx catalyst (a) as prepared and (b) after CO_2 hydrogenation. PXRD pattern of the as prepared FeOx catalyst (a) showed two main diffraction signals appear at 39.7° and 74.4° that support the formation of the pure hematite phase (Fe_2O_3).

3. Stability test of the Cu/FeOx catalyst for CO_2 hydrogenation

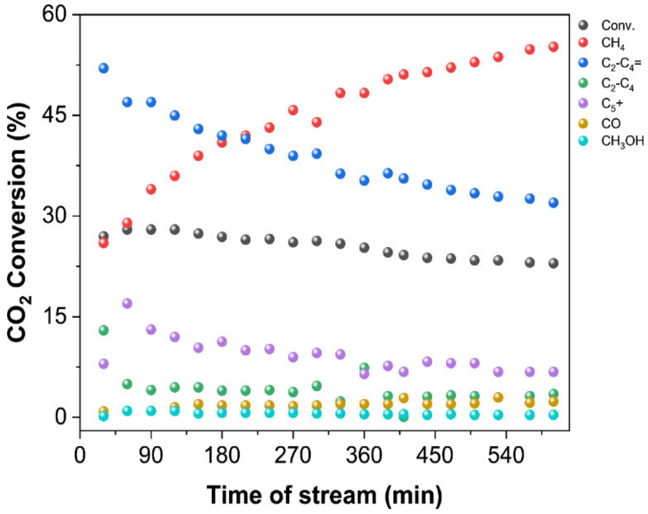

Figure S3. Time on stream test of the Cu/FeOx catalyst at 340 °C. Cat. 200 mg, CO_2/H_2/He (1/4/3.3, total flow 25 ml/min).

4. SEM images of the Cu/FeOx catalyst after CO_2 hydrogenation

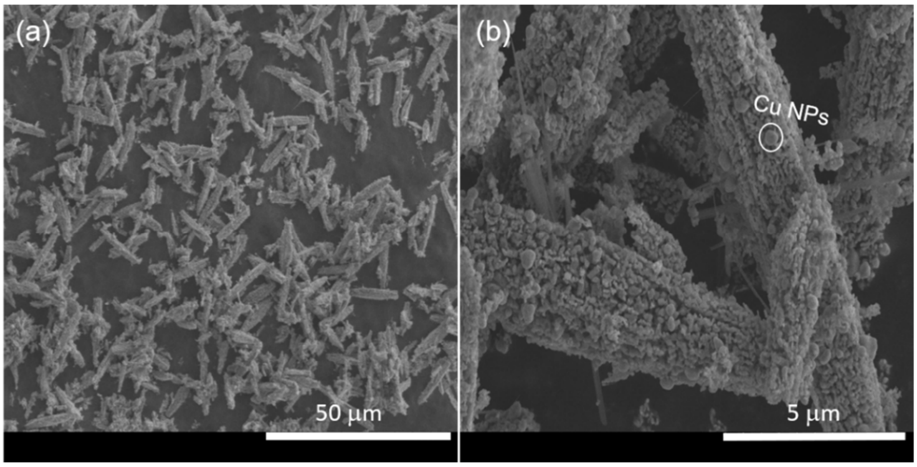

Figure S4. SEM images (a,b) of the spent Cu/FeOx catalys. The spent catalyst preserved it rod like morphology.

5. TEM images of the FeOx catalyst before and after CO_2 hydrogenation

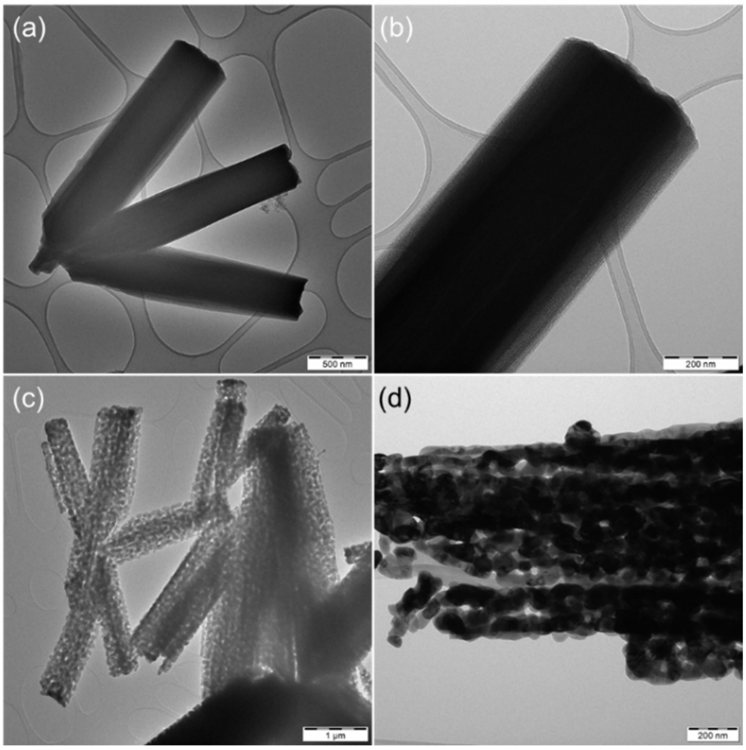

Figure S5. TEM images of as (a,b) prepared FeOx and (c,d) spent FeOx catalyst

6. Literature comparison of CO_2 hydrogenation to hydrocarbons by different Fe-based catalysts

Table S1. Literature review of Fe-based catalysts for CO_2 hydrogenation

Entry	Catalyst	Temp. (°C)	CO_2 Conv. (%)	Selectivity (%)						Ref.
				CH_4	CO	C_2-$C_4^=$	C_2-C_4	C_5+ and others	O/P ratio	
**1**	**Cu/FeOx**	**310**	**18**	**22**	**1**	**40**	**14**	**22**	**2.86**	**this study**
**2**	**Cu/FeOx**	**340**	**27**	**26**	**1**	**52**	**13**	**8**	**0.31**	**this study**
**3**	**Cu/FeOx**	**370**	**32**	**42**	**2**	**43**	**6**	**6**	**7.17**	**this study**
4	K-FeC/ZrO_2	220	40	13	32	25	-	30	-	[1]
5	Na-$CoFe_3O_4$/CNT	340	34.4	14.8	18.6	38.8	5.5	40	7.05	[2]
6	Na-Fe_3O_4	320	40.5	15.8	13.5	46.6	7.5	30.1	6.21	[3]
7	Fe_2O_3	300	23	14	n.a	-	-	65	-	[4]
8	Fe-Cu-K-Al	300	41	15.6	2.5	-	38	44	-	[5]
9	Fe-K/alumina	300	31.3	11	7	-	36	46	-	[6]
10	Fe-Cex	300	25	38	19	-	38	4	-	[7]
11	Fe-La-Cu-K/Al_2O_3	300	32	20	15	-	39	26	-	[8]
12	Fe-Ru-Mn-K/Al_2O_3	300	30	19	18	-	39	25	-	[8]
13	Fe-Ru-K	300	47	16.4	3.1	19.7	7.4	53	2.66	[9]
14	Mn-Na-Fe	320	38.6	11.8	11.8	30.2	4	42.1	7.55	[10]
15	Ru-FeOx/Al_2O_3	300	18	55	-	-	45	-	-	[11]
16	Fe/CNT	350	20	36	22	-	33	9	-	[12]
17	Mn-FeO	340	30	40	7	-	52	n.a	-	[13]
18	Na–Fe_3O_4/HMCM	320	26	8	17	-	26	57	-	[14]
19	Cu/Fe_2O_3	300	17	23	31	-	32	65	-	[15]
20	$ZnFe_2$O4	340	28	44	22	-	34	-	-	[16]
21	Na/FeOx*	320	36	8	10	64	18	-	3.55	[17]
22	Mn/Fe3O4-EDA	320	20	40	41	1	36	23	0.03	[18]
23	Co-Fe	270	23	31	53	-	12	-	-	[19]
24	K-Fe_2O_3	270	36	20	13	35	8	24	4.37	[20]
25	Fe–Co/Al2O3-K	300	36	16	13	-	71	-	-	[21]
26	Fe/Co-Y	300	29.6	14	21	-	30	55	-	[17]
27	Fe-Cu-K	300	29.7	7	17	22	-	54	-	[22]
28	RuFe NPs	175	12	8	-	-	4	88	-	[23]
29	Fe-Mn-K	300	38.2	10.4	5.6	25.2	2	61.9	12.6	[24]
30	Cu/FeOx	370	23.5	60.5	2.5	29	5.7	2.3	-	[25]

* 3 MPa pressure applied

7. Morphology and XRD comparison of the composition of the Cu/FeOx catalyst with previously reported catalysts

Catalysts in this study *Previously reported catalysts*[25]

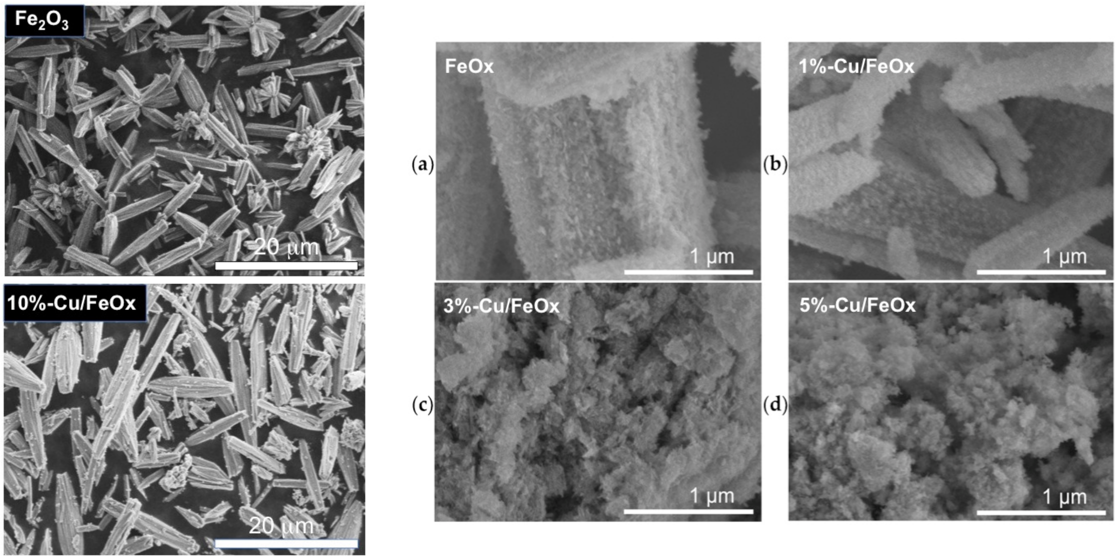

Figure S6. SEM images of the studied *FeOx and 12%-Cu/FeOx* catalysts (left), and previously reported (a) FeO_x, (b) 1%-Cu/FeO_x, (c) 3%-Cu/FeO_x, and (d) 5%-Cu/FeO_x catalysts (right).

Catalyst in this study
(12%-Cu/FeOx)

Previously reported catalyst
(5%-Cu/FeOx)[25]

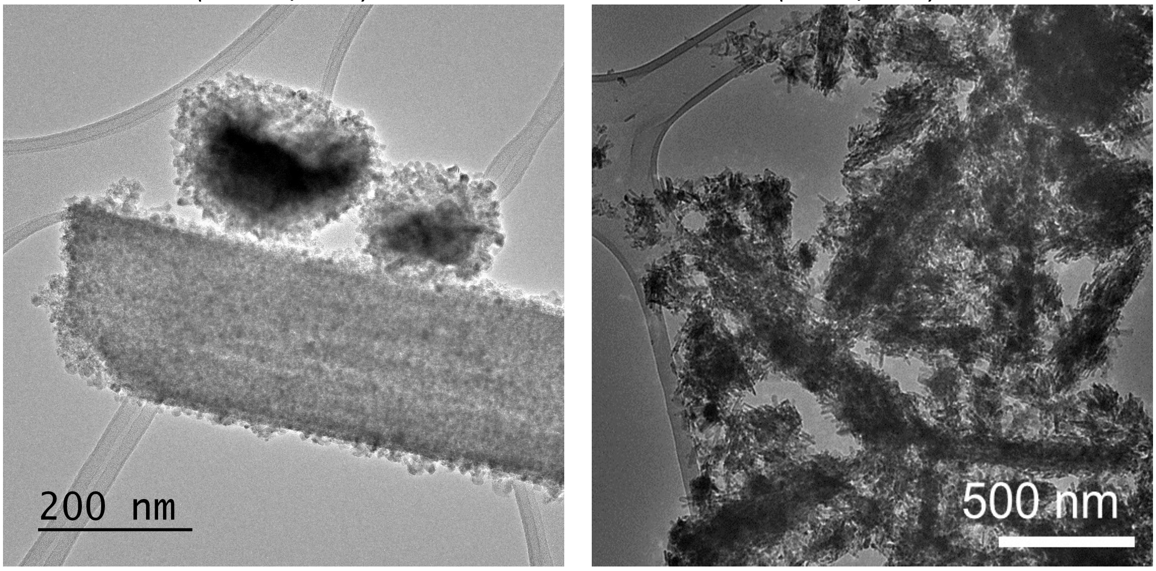

Figure S7. TEM images of the studied *12%-Cu/FeOx* catalyst (left) and previously reported 5%-Cu/FeO_x catalyst (right).

Table S2. XRD analyses comparison of the studied catalysts with previously reported ones

Entry	Catalyst	Iron-composition (%)		
		α-Fe_2O_3	α-FeOOH	γ-Fe
1[25]	FeO_x	43.5	54.5	-
2[25]	1%-Cu/FeO_x	81.2	16.8	-
3[25]	3%-Cu/FeO_x	43.6	56.4	-
4[25]	5%-Cu/FeO_x	48.3	51.7	-
**this study**	**Fe_2O_3**	**100**	**-**	**-**
**this study**	**12%-Cu/FeO_x**	**81.6**	**3.7**	**14.7**

8. References

[1] J. Zhu, G. Zhang, W. Li, X. Zhang, F. Ding, C. Song, X. Guo, Deconvolution of the Particle Size Effect on CO2 Hydrogenation over Iron-Based Catalysts, ACS Catalysis, 10 (2020) 7424-7433.

[2] K.Y. Kim, H. Lee, W.Y. Noh, J. Shin, S.J. Han, S.K. Kim, K. An, J.S. Lee, Cobalt Ferrite Nanoparticles to Form a Catalytic Co–Fe Alloy Carbide Phase for Selective CO2 Hydrogenation to Light Olefins, ACS Catalysis, (2020) 8660-8671.

[3] J. Wei, J. Sun, Z. Wen, C. Fang, Q. Ge, H. Xu, New insights into the effect of sodium on Fe3O4- based nanocatalysts for CO2 hydrogenation to light olefins, Catalysis Science & Technology, 6 (2016) 4786-4793.

[4] M. Albrecht, U. Rodemerck, M. Schneider, M. Bröring, D. Baabe, E.V. Kondratenko, Unexpectedly efficient CO2 hydrogenation to higher hydrocarbons over non-doped Fe2O3, Applied Catalysis B: Environmental, 204 (2017) 119-126.

[5] J.-S. Hong, J.S. Hwang, K.-W. Jun, J.C. Sur, K.-W. Lee, Deactivation study on a coprecipitated Fe-Cu-K-Al catalyst in CO2 hydrogenation, Applied Catalysis A: General, 218 (2001) 53-59.

[6] J.S. Hwang, K.-W. Jun, K.-W. Lee, Deactivation and regeneration of Fe-K/alumina catalyst in CO2 hydrogenation, Applied Catalysis A: General, 208 (2001) 217-222.

[7] F.J. Pérez-Alonso, M. Ojeda, T. Herranz, S. Rojas, J.M. González-Carballo, P. Terreros, J.L.G. Fierro, Carbon dioxide hydrogenation over Fe–Ce catalysts, Catalysis Communications, 9 (2008) 1945-1948.

[8] U. Rodemerck, M. Holeňa, E. Wagner, Q. Smejkal, A. Barkschat, M. Baerns, Catalyst Development for CO2 Hydrogenation to Fuels, ChemCatChem, 5 (2013) 1948-1955.

[9] F. Jiang, B. Liu, S. Geng, Y. Xu, X. Liu, Hydrogenation of CO2 into hydrocarbons: enhanced catalytic activity over Fe-based Fischer–Tropsch catalysts, Catalysis Science & Technology, 8 (2018) 4097-4107.

[10] B. Liang, T. Sun, J. Ma, H. Duan, L. Li, X. Yang, Y. Zhang, X. Su, Y. Huang, T. Zhang, Mn decorated Na/Fe catalysts for CO2 hydrogenation to light olefins, Catalysis Science & Technology, 9 (2019) 456-464.

[11] A. Aitbekova, E.D. Goodman, L. Wu, A. Boubnov, A.S. Hoffman, A. Genc, H. Cheng, L. Casalena, S.R. Bare, M. Cargnello, Engineering of Ruthenium–Iron Oxide Colloidal Heterostructures: Improved Yields in CO2 Hydrogenation to Hydrocarbons, Angewandte Chemie International Edition, 58 (2019) 17451-17457.

[12] S.A. Chernyak, A.S. Ivanov, D.N. Stolbov, S.V. Maksimov, K.I. Maslakov, P.A. Chernavskii, Y.A. Pokusaeva, A.E. Koklin, V.I. Bogdan, S.V. Savilov, Sintered Fe/CNT framework catalysts for CO2 hydrogenation into hydrocarbons, Carbon, (2020).

[13] M. Al-Dossary, A.A. Ismail, J.L.G. Fierro, H. Bouzid, S.A. Al-Sayari, Effect of Mn loading onto MnFeO nanocomposites for the CO2 hydrogenation reaction, Applied Catalysis B: Environmental, 165 (2015) 651-660.

[14] J. Wei, R. Yao, Q. Ge, Z. Wen, X. Ji, C. Fang, J. Zhang, H. Xu, J. Sun, Catalytic Hydrogenation of CO2 to Isoparaffins over Fe-Based Multifunctional Catalysts, ACS Catalysis, 8 (2018) 9958-9967.
[15] Y.H. Choi, Y.J. Jang, H. Park, W.Y. Kim, Y.H. Lee, S.H. Choi, J.S. Lee, Carbon dioxide Fischer-Tropsch synthesis: A new path to carbon-neutral fuels, Applied Catalysis B: Environmental, 202 (2017) 605-610.
[16] Y.H. Choi, E.C. Ra, E.H. Kim, K.Y. Kim, Y.J. Jang, K.-N. Kang, S.H. Choi, J.-H. Jang, J.S. Lee, Sodium-Containing Spinel Zinc Ferrite as a Catalyst Precursor for the Selective Synthesis of Liquid Hydrocarbon Fuels, ChemSusChem, 10 (2017) 4764-4770.
[17] B. Liang, H. Duan, T. Sun, J. Ma, X. Liu, J. Xu, X. Su, Y. Huang, T. Zhang, Effect of Na Promoter on Fe-Based Catalyst for CO2 Hydrogenation to Alkenes, ACS Sustainable Chemistry & Engineering, 7 (2019) 925-932.
[18] B. Liu, S. Geng, J. Zheng, X. Jia, F. Jiang, X. Liu, Unravelling the New Roles of Na and Mn Promoter in CO2 Hydrogenation over Fe3O4-Based Catalysts for Enhanced Selectivity to Light α-Olefins, ChemCatChem, 10 (2018) 4718-4732.
[19] M.K. Gnanamani, G. Jacobs, H.H. Hamdeh, W.D. Shafer, F. Liu, S.D. Hopps, G.A. Thomas, B.H. Davis, Hydrogenation of Carbon Dioxide over Co–Fe Bimetallic Catalysts, ACS Catalysis, 6 (2016) 913-927.
[20] C.G. Visconti, M. Martinelli, L. Falbo, A. Infantes-Molina, L. Lietti, P. Forzatti, G. Iaquaniello, E. Palo, B. Picutti, F. Brignoli, CO2 hydrogenation to lower olefins on a high surface area K-promoted bulk Fe-catalyst, Applied Catalysis B: Environmental, 200 (2017) 530-542.
[21] R. Satthawong, N. Koizumi, C. Song, P. Prasassarakich, Bimetallic Fe–Co catalysts for CO2 hydrogenation to higher hydrocarbons, Journal of CO2 Utilization, 3-4 (2013) 102-106.
[22] W. Wang, X. Jiang, X. Wang, C. Song, Fe–Cu Bimetallic Catalysts for Selective CO2 Hydrogenation to Olefin-Rich C2+ Hydrocarbons, Industrial & Engineering Chemistry Research, 57 (2018) 4535-4542.
[23] M.I. Qadir, A. Weilhard, J.A. Fernandes, I. de Pedro, B.J.C. Vieira, J.C. Waerenborgh, J. Dupont, Selective Carbon Dioxide Hydrogenation Driven by Ferromagnetic RuFe Nanoparticles in Ionic Liquids, ACS Catalysis, 8 (2018) 1621-1627.
[24] B. Yao, T. Xiao, O.A. Makgae, X. Jie, S. Gonzalez-Cortes, S. Guan, A.I. Kirkland, J.R. Dilworth, H.A. Al-Megren, S.M. Alshihri, P.J. Dobson, G.P. Owen, J.M. Thomas, P.P. Edwards, Transforming carbon dioxide into jet fuel using an organic combustion-synthesized Fe-Mn-K catalyst, Nature Communications, 11 (2020) 6395.
[25] K.Q. Simkovičová, M.I. Qadir; Žilková, N.; Olszówka, J.E.; Sialini, P.; Kvítek, L.; Vajda, Š., Hydrogenation of CO_2 on Nanostructured Cu/FeOx Catalysts: The Effect of Morphology and Cu Load on Selectivity., Catalysts 12 (2022) 516.
